# Comparative genomics of cocci-shaped *Sporosarcina* strains with diverse spatial isolation

**DOI:** 10.1186/s12864-018-4635-8

**Published:** 2018-05-02

**Authors:** Andrew Oliver, Matthew Kay, Kerry K. Cooper

**Affiliations:** 1 Department of Biology, California State University Northridge, Northridge, CA USA; 2 School of Animal and Comparative Biomedical Sciences, University of Arizona, Tucson, AZ USA; 3 Present Address: Molecular Biology and Biochemistry, University of California Irvine, Irvine, CA USA

**Keywords:** *Sporosarcina ureae*, Cocci, Spore-forming, Comparative genomics, *Sporosarcina*

## Abstract

**Background:**

Cocci-shaped *Sporosarcina* strains are currently one of the few known cocci-shaped spore-forming bacteria, yet we know very little about the genomics. The goal of this study is to utilize comparative genomics to investigate the diversity of cocci-shaped *Sporosarcina* strains that differ in their geographical isolation and show different nutritional requirements.

**Results:**

For this study, we sequenced 28 genomes of cocci-shaped *Sporosarcina* strains isolated from 13 different locations around the world. We generated the first six complete genomes and methylomes utilizing PacBio sequencing, and an additional 22 draft genomes using Illumina sequencing. Genomic analysis revealed that cocci-shaped *Sporosarcina* strains contained an average genome of 3.3 Mb comprised of 3222 CDS, 54 tRNAs and 6 rRNAs, while only two strains contained plasmids. The cocci-shaped *Sporosarcina* genome on average contained 2.3 prophages and 15.6 IS elements, while methylome analysis supported the diversity of these strains as only one of 31 methylation motifs were shared under identical growth conditions. Analysis with a 90% identity cut-off revealed 221 core genes or ~ 7% of the genome, while a 30% identity cut-off generated a pan-genome of 8610 genes. The phylogenetic relationship of the cocci-shaped *Sporosarcina* strains based on either core genes, accessory genes or spore-related genes consistently resulted in the 29 strains being divided into eight clades.

**Conclusions:**

This study begins to unravel the phylogenetic relationship of cocci-shaped *Sporosarcina* strains, and the comparative genomics of these strains supports identification of several new species.

**Electronic supplementary material:**

The online version of this article (10.1186/s12864-018-4635-8) contains supplementary material, which is available to authorized users.

## Background

Sporulation is a crucial survival mechanism for many types of bacteria, which can allow spore-forming bacteria to colonize and/or survive in very diverse environments. Oddly, very few spore-forming cocci have been identified or characterized, and the overall knowledge of cocci-shaped spore-formers is very limited at best. All six described species are Gram-positive, but three were actually designated as coccobacilli [1, 2] or coccoid [3] and have undergone several reclassifications [4, 5]. In fact, *Halobacillus halophilis* was originally described as coccoid is now referred to as a bacillus [6]. Three cocci-shaped spore-forming species have been characterized, including two anaerobic species (*Sarcina ventriculi* and *Sarcina maxima*) [7–9] and one aerobic bacteria (*Sporosarcina ureae*) [10], but the genomics of the different species have not been investigated, particularly *Sporosarcina ureae* strains*.*

Currently, the genus *Sporosarcina* is composed almost entirely of bacilli-shaped species [11], while *S. ureae* is the only established cocci-shaped *Sporosarcina*. To date, the only analysis surveying the geographic and physiological diversity of *S. ureae* or any cocci-shaped *Sporosarcina* strains comes from work done by Bernadine Pregerson over 40 years ago. During the study, over 50 isolates of cocci-shaped *Sporosarcina* strains were collected from numerous locations around the world, including four different continents. Pregerson originally identified each isolate as *S. ureae* based on cell morphology, cell arrangement and spore-forming ability, and examined nutritional requirements necessary for growth. The study indicated four major nutritional requirement groups, but failed to reveal any correlation between nutritional requirements and habitat [12]. In 1996, Risen studied the electrophoretic mobilities of 24 metabolic enzymes of these cocci-shaped *Sporosarcina* strains from Pregerson’s study, and concluded that these strains were non-clonal [13]. We hypothesize that based on the studies of Pregerson and Risen, there are novel species of cocci-shaped *Sporosarcina*. However, the high resolution of whole genome sequencing (WGS) is required to accurately unravel the diversity of these cocci-shaped *Sporosarcina* strains.

The goal of this study was to investigate the diversity of these cocci-shaped *Sporosarcina* strains at the genomic level utilizing next-generation sequencing, and further our understanding of the phylogenetic relationship of the genus *Sporosarcina.* The study is particularly novel as virtually nothing is known about the overall genomics of the genus especially cocci-shaped *Sporosarcina* strains. To date the only cocci-shaped *Sporosarcina* genome is a single draft genome of *S. ureae* (strain DSM 2281, Genbank Accession: NZ_AUDQ00000000), and there has been no analysis or research utilizing this genome. For this study, we sequenced the genomes of 28 cocci-shaped *Sporosarcina* strains isolated from 13 different locations around the world, with at least one representative of each of Pregerson’s four original nutritional growth requirement groups [12]. Comparative genomics of cocci-shaped *Sporosarcina* strains will assist in resolving the diversity of these strains, while also providing a future genetic resource for investigating processes such as sporulation in cocci-shaped bacteria.

Examining the genomics of phenotypically different and geographically diverse cocci-shaped *Sporosarcina* strains; we found an average genome size of 3.3 Mb, which encoded for 3222 CDS, 54 tRNAs and 6 rRNAs. Examination of spore genes found the cocci-shaped *Sporosarcina* strains were lacking many genes that are found in *Bacillus*; however, spore genes that are present are well conserved among all the strains. In-depth genomic analysis of the strains demonstrated a highly diverse group, as comparative genomic analysis using a 90% identity cut-off revealed 221 core genes or ~ 7% of the genome, while a 30% identity cut-off generated a pan-genome of 8610 genes. Methylome analysis also supported the diversity, as there were numerous different adenine and cytosine methylation motifs, but only one motif was shared between two of six strains grown under identical conditions. Both core and accessory gene diversity failed to correlate with the nutritional growth requirements or location of isolation for the strains. Overall, this study begins to unravel the genomics and phylogenetic relationship of the genus *Sporosarcina*, particularly revealing the genomic diversity of cocci-shaped strains, and indicates there are additional cocci-shaped *Sporosarcina* species. Furthermore, it provides a genetic resource for investigating the sporulation process in cocci-shaped bacteria.

## Methods

### Strains and accession numbers

All strains sequenced in this study were originally identified as *S. ureae* by Pregerson, based on cell morphology, cell arrangement and sporulation ability [12]. All 28 genomes generated during this study are publically available on NCBI by their respective accession numbers (Table 1). Additional analysis performed with genome sequences not generated during this study were obtained from the NCBI Genome database under the following accession numbers: NZ_AUDQ00000000 (*S. ureae* DSM 2281).

**Table 1:** General genomic characteristics of cocci-shaped *Sporosarcina* strains

Accession ID	Strain	Location Isolated	Sequencing platform	Coverage	Contigs	Base pairs (bp)	%GC	CDS	tRNAs	Plasmid(s)	Prophage(s)^a^	Insertion Sequences
PDZF00000000	*Sporosarcina* sp. P7	USA: Woodland Hills, CA	Illumina	4457	38	3,169,294	41.4	3071	55	0	0 (6)	10
PDZE00000000	*Sporosarcina* sp. P3a	USA: Reseda, CA	Illumina	443	34	3,379,590	41.5	3238	50	0	0 (2)	11
PDZD00000000	*Sporosarcina* sp. P35	USA: Honolulu, HI	Illumina	614	75	3,252,669	44.5	3247	59	1	2 (1)	16
PDZC00000000	*Sporosarcina* sp. P34	USA: Waikiki, HI	Illumina	544	23	3,262,144	41.4	3203	44	0	1 (1)	8
PDZB00000000	*Sporosarcina* sp. P32b	Japan	Illumina	506	70	3,314,266	41.3	3218	58	0	0 (2)	15
PDZA00000000	*Sporosarcina* sp. P31	Japan	Illumina	479	70	3,316,673	41.3	3218	57	0	0 (2)	17
PDYZ00000000	*Sporosarcina* sp. P30	Japan	Illumina	482	72	3,313,921	41.3	3215	58	0	0 (2)	15
PDYY00000000	*Sporosarcina* sp. P2a	USA: Canoga Park, CA	Illumina	573	33	3,328,642	41.3	3235	52	0	0 (1)	12
PDYX00000000	*Sporosarcina* sp. P29	Japan: Yokahama	Illumina	531	160	3,382,122	41.4	3325	58	0	0 (2)	19
PDYW00000000	*Sporosarcina* sp. P26b	Japan: Tokyo	Illumina	501	66	3,382,782	41.2	3246	50	0	0 (2)	11
PDYV00000000	*Sporosarcina* sp. P25	Japan: Tokyo	Illumina	572	45	3,269,093	41.1	3182	51	0	0 (3)	13
PDYU00000000	*Sporosarcina* sp. P21c	USA: Berkeley, CA	Illumina	442	50	3,384,350	42.2	3292	54	0	1 (0)	13
PDYT00000000	*Sporosarcina* sp. P20a	USA: Berkeley, CA	Illumina	515	58	3,314,917	41.4	3228	56	0	0 (1)	12
PDYS00000000	*Sporosarcina* sp. P1a	USA: Canoga Park, CA	Illumina	866	48	3,306,654	41.2	3194	50	0	0 (2)	13
PDYR00000000	*Sporosarcina* sp. P19	USA: Berkeley, CA	Illumina	504	34	3,339,001	41.4	3216	50	0	0 (3)	5
PDYQ00000000	*Sporosarcina* sp. P18a	USA: Berkeley, CA	Illumina	559	18	3,219,274	41.4	3149	51	0	1 (1)	4
PDYP00000000	*Sporosarcina* sp. P17b	USA: Berkeley, CA	Illumina	529	52	3,401,750	41.3	3336	60	0	1 (5)	11
PDYO00000000	*Sporosarcina* sp. P16b	USA: Berkeley, CA	Illumina	475	44	3,363,873	41.1	3281	51	0	0 (1)	19
PDYN00000000	*Sporosarcina* sp. P16a	USA: Berkeley, CA	Illumina	427	46	3,293,369	41.1	3185	56	0	0 (3)	12
PDYM00000000	*Sporosarcina* sp. P13	USA: San Diego, CA	Illumina	800	85	3,318,423	40.6	3234	54	0	1 (2)	16
PDYL00000000	*Sporosarcina* sp. P12	USA: San Diego, CA	Illumina	539	42	3,438,859	41.4	3372	56	0	0 (1)	16
PDYK00000000	*Sporosarcina* sp. P10	USA: San Diego, CA	Illumina	510	43	3,437,370	41.4	3374	47	0	0 (1)	15
CP015108	*Sporosarcina ureae* str. S204	South Africa: Pretoria	Pacific Biosciences	174	1	3,362,333	41.5	3196	60	0	0 (1)	46
CP015027	*Sporosarcina* sp. P33	Japan: Tokyo	Pacific Biosciences	158	1	3235,441	44.5	3050	68	0	1 (1)	29
CP015109	*Sporosarcina* sp. P17a	USA: Berkeley, CA	Pacific Biosciences	183	1	3,412,428	41.5	3204	68	0	0 (2)	15
CP015207	*Sporosarcin*a sp. P8	USA: Los Angeles, CA	Pacific Biosciences	707	1	3,353,765	41.2	3164	69	0	0 (3)	12
CP015348	*Sporosarcina* sp. P32a	Japan	Pacific Biosciences	169	1	3,382,744	41.4	3183	68	0	0 (4)	28
CP015349	*Sporosarcina* sp. P37	USA: Boston, MA	Pacific Biosciences	150	1	3,271,521	44.7	3155	68	1	2 (1)	37
NZ_AUDQ00000000	*Sporosarcina ureae* str. DSM 2281	unknown (Type Strain)	Illumina	unknown	36	3,318,232	41.4	3241	56	0	0 (2)	2

^a^Numbers outside parentheses are complete prophages, while number within are total number of prophages

### DNA extraction

DNA was extracted as described by Miller et al. [14] with several modifications. Each cocci-shaped *Sporosarcina* strain was grown up in triplicate in 5 ml of tryptic soy yeast broth (27.5 g Tryptic Soy Broth, 5 g Yeast Extract; Fisher Scientific, Fairlawn, New Jersey, USA) on a rotator at 30 °C overnight. The replicates were then combined, pelleted (10 min @ 12,000 x g), re-suspended in 1.5 ml Tris-sucrose (10% sucrose; Fisher Scientific; 50 mM Tris, pH 8.0; Research Organics Cleveland, Ohio, USA) and diluted to an optical density of 1.6-1.8. Cells were lysed with 500 μl lysozyme (20 mg/ml in 50 mM Tris, pH 8.0; Fisher Scientific) and 300 μl 10% SDS, and then 600 μl EDTA (100 mM EDTA, pH 8.0; Fisher Scientific) was used to buffer the suspended DNA. Twenty μl RNase (10 mg/ml; Fisher Scientific) was added and incubated at 37 °C for 24 h to ensure total RNA removal. Next, 10 μl proteinase K (20 mg/ml; Fisher BioReagents) was added and incubated at 37 °C for 4 h to remove any remaining proteins, and then 265 μl 3 M sodium acetate (pH 5.5; Fisher Scientific) plus 6 ml absolute ethanol were added to precipitate the DNA. Precipitated DNA was transferred and re-suspended in 400 μl EB buffer (Qiagen) by incubating at 37 °C overnight. Next, 400 μl of phenol:chloroform:isoamyl alcohol (Fisher BioReagents) was added, followed by separation via centrifugation (12,000 x g; 5 min), and the aqueous layer transferred. To remove any traces of phenol from the solution, 400 μl of chloroform (Fisher Scientific) was added and mixed by inverting three times, centrifuged (12,000 x g; 3 min), the top aqueous layer transferred to a new tube, and the DNA precipitated again with absolute ethanol. The precipitated DNA was transferred and again re-suspended in 200 μl EB buffer by incubating at 37 °C overnight. Quality, size and quantity of DNA were confirmed with a Nanodrop spectrophotometer (260/280 = 1.8-2.0), gel electrophoresis (high single band, little smearing) and a Picogreen dsDNA assay (Life Technologies’ Quant-iT Picogreen dsDNA kit) per the manufacturer’s instructions, respectively.

### Pacific Biosciences (PacBio) sequencing

Extracted DNA for six cocci-shaped *Sporosarcina* strains were sent to the UC Irvine Genomic High Throughput Facility for library preparation and PacBio sequencing. Library preparation involved shearing 15 μg of genomic DNA using Covaris G-Tubes, according to the manufactures instructions, resulting in 20 kb fragments used for generating the PacBio sequencing libraries. Blue Pippen (Sage Science) was used to select DNA fragments of 8 kb-50 kb length. Library and sequencing kits used SMRTbell Template Prep Kit (v1.0), DNA Polymerase binding kit P6, and DNA Sequencing Reagent (v4.0), and a 100pM-125pM concentration was loaded onto the SMRT cell. One SMRT cell/strain allowed for > 150× coverage per strain, ample coverage for the construction of de novo genomes. The sequencing run lasted 4 h for each strain. In total, the six strains resulted in an average of 67,798 reads, an average read length of 13.57 kb, and an average of 166× coverage per genome (Table 1).

### Illumina sequencing

Fifteen μg of genomic DNA from each of the 22 cocci-shaped *Sporosarcina* strains were sheared into 400 bp fragments using a Covaris ME220 Focused Acoustic Shearer per the manufacturer’s recommended protocol. Barcoded Illumina sequencing libraries were prepared from the sheared fragments using the NEBNext Multiplex Oligos for Illumina (96 Index Primers; New England BioLabs, Ipswich, MA, USA) and NEBNext Ultra II DNA Library Preparation Kit for Illumina (New England BioLabs) following the manufacturer’s instructions. Barcoded libraries were quality checked using an Experion Automated Electrophoresis System (Bio-Rad, Hercules, CA, USA), quantified using a Picogreen dsDNA assay, and then pooled in equal molar ratios for sequencing. The pooled sequencing libraries were then sent to GeneWiz (South Plainfield, NJ, USA) for paired-end Illumina sequencing (2 × 150 bp) on an Illumina HiSeq X machine. In total, sequencing the 22 strains resulted in a median of 11.7 million reads and 522× coverage per genome (Table 1).

### Assembly and annotation

PacBio genomes were assembled using SMRTanalysis software (v2.3.0.1), and any genomes that needed further assembly were done in silico by using Geneious software (Biomatters, v9.0.0) to map the corrected reads to the contigs and subsequently linking the contigs into a complete genome sequence [15].

The raw Illumina sequence reads for each of the strains were examined using Fastqc [16] and quality filtered using Prinseq [17] using parameters (min_qual_mean 39, ns_max_n 0) to select for high quality reads. Reads were sub-sampled to roughly 80× coverage (based on a genome size of 3.3 Mb) and assembled with the a5 assembler [18] using default parameters.

All genomes were annotated using NCBI’s Prokaryote Genome Automatic Annotation Pipeline (PGAAP). Reverse Position Specific-BLAST (RPS-BLAST) was used to find the Cluster of Orthologous Groups (COG) data for each of the genomes. Briefly, each set of query proteins were BLASTed against NCBI’s Conserved Domain Database (CDD) using RPS-BLAST [19]. CDD contains well-annotated multiple sequence alignment models for ancient domains and full-length proteins, allowing for fast identification of conserved domains in the query proteins. After matching the query proteins to the CDD proteins with RPS-BLAST, a perl script [20] was used to pair the correct COG information to each matched query protein. NCBI provides the COG data for each of the genomes contained in the CDD, and this was cross-referenced with the BLAST results to obtain COG information for the query proteins.

### 16S rRNA analysis

After annotation, each PacBio complete genome was BLASTed, using the BLAST plugin in Geneious, with a copy of its own 16S rRNA gene to verify that the genomes did not contain unidentified rRNA loci or genes. Due to small known variation between copies of the gene, even within the same genome, a consensus sequence of all copies of the gene within the genome helps capture the most accurate single sequence to use in a comparative analysis [21]. Therefore, for each of the six strains that were sequenced with PacBio, we created a strain specific consensus sequence for the 16S rRNA gene from the different copies of the gene throughout the genome.

The 16S gene in the Illumina-sequenced draft genomes was predicted using Barrnap (http://www.vicbioinformatics.com/software.barrnap.shtml), and 198 genus *Sporosarcina* 16S rRNA gene sequences were downloaded from the Ribosomal Database Project (RDP) [22]. The sequences were aligned using SILVA Incremental Aligner (SINA, www.arb-silva.de) an alignment tool that takes into account ribosomal secondary structure when aligning sequences [23]. FastTree2 was used to build a phylogenetic tree using GTR + gamma20 parameters and 1000 bootstrap replicates [24]. Tree visualization was done using the web-based program interactive Tree of Life (iTOL) [25].

### Geographic distribution analysis

To investigate the geographic distribution of the genus *Sporosarcina*, location data was gathered from the Earth Microbiome Project (EMP) [26] and RDP [22]. Data was extracted from the EMP using the Redbiom tool (https://github.com/biocore/redbiom), which queried the database for the genus *Sporosarcina* across all available contexts. Sample metadata from matching features returned were parsed for latitude and longitude data. Data from the RDP was downloaded in Genbank format, and the location information (generally City/Region field) as queried against the OpenStreetMap Nomatim (nominatim.openstreetmap.org) database to obtain approximate latitude and longitude. The data from both sources were then categorized based on general sample type into one of four groups; environment, animal, plant, or human. A map was created using the Matplotlib and Basemap packages in Python, with rendering using GEOS (Geometry Engine - Open Source).

### Pan/Core genome analysis

There are currently no standard parameters to elucidate the core genome of related species, therefore we used the following core genome parameters (percent amino acid sequence identity (PI), percent query coverage (PC), and E-value), and set those cutoffs to the strict values of > 90% PI, > 90% PC and > 1e^− 4^ E-value [27]. To determine the pan genome, we used established sequence parameters (30% PI) [28] to identify orthologous gene clusters. Any cluster generated in this step that was unique to a strain was identified as a strain-specific gene.

To generate the core genome sequence comparison data, we created a protein BLAST database of all protein sequences from the 28 sequenced genomes and the downloaded *S. ureae* DSM 2281 genome. Next, the protein sequences were individually compared for each genome using the BLASTp command from the BLAST+ software [29] against the created protein BLAST database. The output showed if the gene in the query genome were present in all the other database genomes, and how related they were to each other. This resulted in a raw data file for each genome that would contribute to the core genome. A large dataset is generated in the previous step and a python program called Geneparser (https://github.com/mmmckay/geneparser) was written to parse the files and identify core/pan/strain-specific genes present in each genome. Geneparser uses the organism specific amino acid sequence files and generates concatenated gene sequences of all the shared genes, where all shared genes are placed in the same order for each genome. The concatenated sequences were aligned with MAFFT using the default settings [30]. Using the resulting alignment file, a phylogenetic tree was constructed with FastTree [31] using JTT + CAT parameters and 1000 bootstrap replicates.

### Methylome analysis

Previously described SMRTanalysis software was used to identify any base modifications by identifying locations of methylation associated with different motifs between all six PacBio sequenced genomes. Additionally, each motif was run through the REBASE database (http://rebase.neb.com/rebase/rebase.html, New England Biolabs) to check if the motifs were associated with any known restriction enzymes and their associated organisms. Only methylation sites that have a Phred-like Quality Value (QV) score of 50 or greater were presented in this study [32]. To visualize the methylomes, all modifications were plotted against each genome using Circos (v.0.69) [33].

### Synteny analysis

Contigs for each of the draft genomes (Illumina sequenced strains and *S. ureae* str. DSM 2281) were re-ordered using the program Mauve (v2.4.0) [34], with the closest related strain with a complete genome utilized as the reference genome. Next, Artemis Comparison Tool (ACT) comparison files were generated between two targeted genomes for comparison by using the blastall command from BLAST+ software, and this was repeated for all 29 cocci-shaped *Sporosarcina* genomes. Finally, the alignments between the different genomes were generated and visualized using the program ACT (v13.0.0) [35].

### Mobile genetic element analysis

Potential prophage sequences in the genome were identified and categorized (intact, incomplete or questionable) using the PHASTER website (http://phaster.ca/) [36]. Insertion sequences located within the genome were identified using the ISfinder website (https://www-is.biotoul.fr/) [36] using a cutoff of 75% identity across 75% of the insertion sequence. Additionally, each of the genomes was manually reviewed for the presence of transposase sequences.

In order to determine the presence of plasmids in the draft genomes, a database of all the < 200 kb size contigs from all the draft genomes was generated. Then the sequence from the plasmid pSporoP37 identified in PacBio sequenced strain P37 was used to identify potential plasmid sequences among the contigs by using the BLASTn command from the BLAST+ software against the database. Finally, each of the < 200 kb size contigs were further examined using BLASTn against the non-redundant (nr) database (https://blast.ncbi.nlm.nih.gov/Blast.cgi), and determining any plasmid sequence hits.

### Whole genome comparison analysis

To compare the average nucleotide identity between each of the 29 cocci-shaped *Sporosarcina* genomes, a BLAST Atlas comparing all the genomes to each strain as the reference genome were generated using the montage project command in the CGView Comparison Tool [37]. To visualize the results, strains with complete genomes were utilized as the reference genomes. The visualized reference strains included S204 that is closely related to the type strain DSM 2281, and P33 that is distantly related to DSM 2281.

The average amino acid identity (AAI) matrix was generated using the Genome-based distance matrix calculator website (http://enve-omics.ce.gatech.edu/g-matrix/), with the default parameters, and the species cutoff value was set at 95% as suggested in Konstantinidis and Tiedje [38].

## Results

### Biogeographical analysis of the genus *Sporosarcina*

**Fig. 1:**
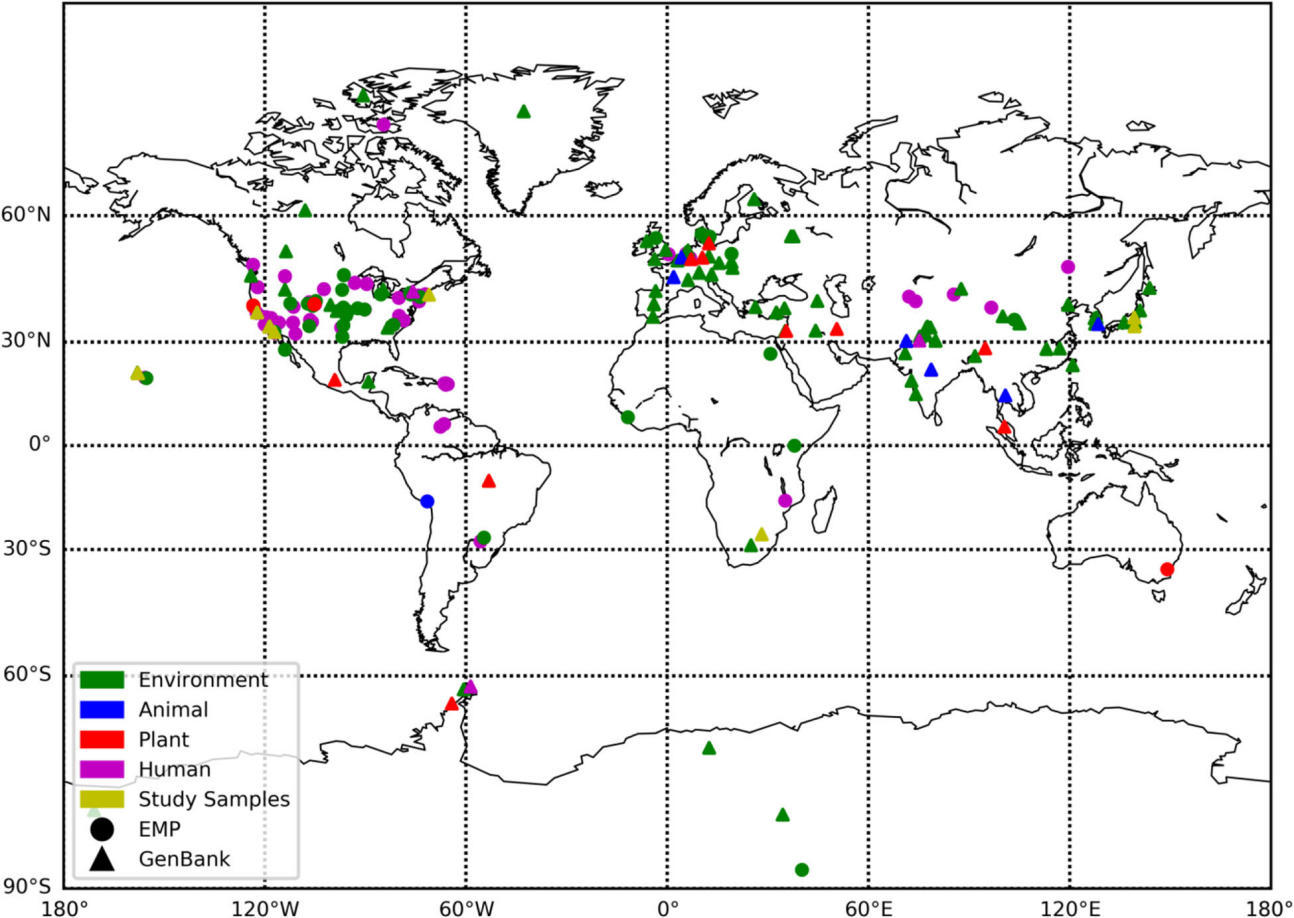
Geographic distribution of the genus *Sporosarcina*, using location data from the Earth Microbiome Project (circles), and Genbank (triangles). Colors indicate the general source of isolation, based on sequence metadata, with the exception of orange that indicates cocci-shaped *Sporosarcina* strains. When exact GPS coordinates were not available, coordinates were approximated based on location data provided. The map was created using the Matplotlib and Basemap packages in Python, with rendering using GEOS (Geometry Engine - Open Source)

As the cocci-shaped *Sporosarcina* strains used in this study were isolated from soil samples from vastly different geographical locations, including three U.S. states on opposite sides of the country (Hawaii, California, and Massachusetts) and three different continents (North America, Africa, and Asia), we examined the most common environments and spatial distribution for the entire genus *Sporosarcina*. Genbank and the EMP revealed that the genus *Sporosarcina* has been found on all seven continents of the world, confirming that not only do cocci-shaped *Sporosarcina* strains have a diverse global distribution, but the entire genus does as a whole (Fig. 1). Additionally, the cocci-shaped *Sporosarcina* strains were all isolated from soil environments, but the analysis of the genus did also find species associated with animals, plants, and humans as well as other environments. However, compiling all the different environments finds that *Sporosarcina* is most commonly associated with terrestrial environments.

### Phylogenetic analysis of the genus *Sporosarcina*

**Fig. 2:**
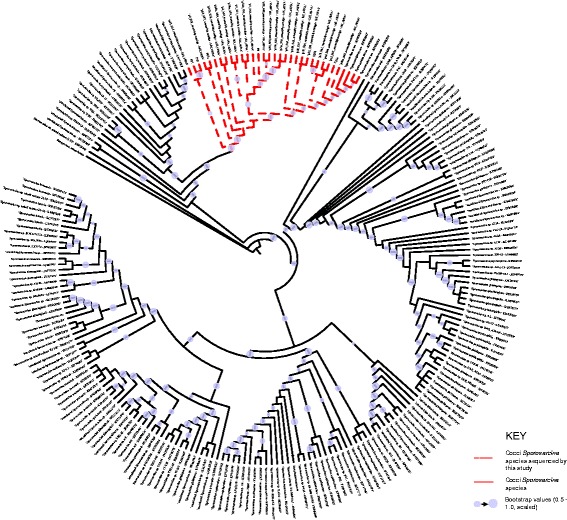
Phylogenetic tree based on all the 16S rRNA gene sequences of the genus *Sporosarcina* deposited on the Ribosomal Database Project. Sequences were filtered to be greater or equal to 1200 bases long, good sequence quality, and both cultured and uncultured organisms. The sequences were aligned using SILVA, and refined using MUSCLE. The tree was built using FastTree and visualized in iTOL

Utilizing public data and the sequences generated during this study (226 16S rRNA gene sequences), we examined the phylogenetic relatedness of the entire genus to begin to understand its diversity. This permitted us to determine the phylogenetic relationship the cocci-shaped *Sporosarcina* strains have to the other species within the genus *Sporosarcina*, and demonstrates exactly where these cocci-shaped strains fit in a genus of mostly bacillus-shaped bacteria. The analysis revealed that the closest bacillus-shaped *Sporosarcina* species to the cocci-shaped strains is *S. newyorkensis*, which at the 16S rRNA gene level share 98.1% pairwise identity with P37, 97.2% pairwise identity with P13, and 96.9% pairwise identity with S204 (Fig. 2). The 28 sequenced cocci-shaped *Sporosarcina* strains clustered together with the few strains of *S. ureae* from the public data; however, there was still quite a bit of diversity within the group as the strains were separated into 11 different clades. For example, P33, P35, and P37 were grouped into clade 1 with 100% pairwise identity with each other, and 99.3% pairwise identity with the next closest relatives, P3 and P17a. However, they only have 97.6% pairwise identity to P13, which is just slightly higher than P13 to *S. newyorkensis*. Plotting pairwise 16S rRNA gene identity against distance between the approximate isolation site failed to show a correlation (*R*^*2*^ = .0002), and overall the 16S rRNA gene analysis found that geographic isolation location was a poor predictor of relatedness of the genus *Sporosarcina* (Additional file 1: Figure S1).

### General genomic characteristics of cocci-shaped *Sporosarcina* strains

**Fig. 3:**
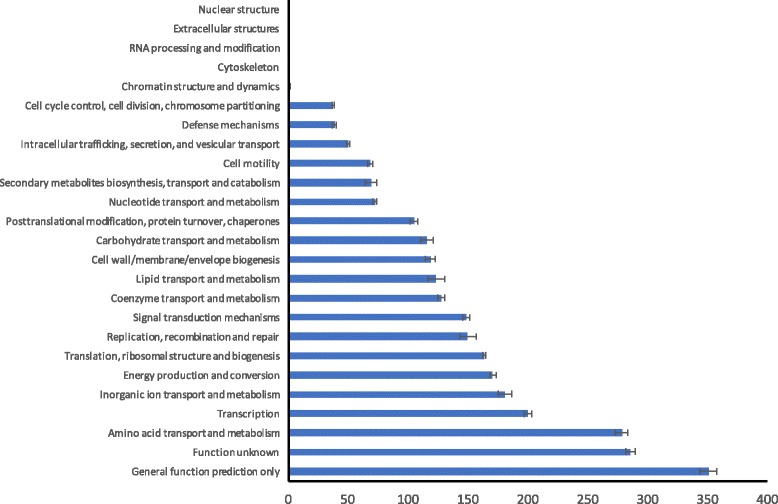
Average number of genes for 29 cocci *Sporosarcina* strains in the different categories of the clusters of orthologous groups (COG) using RPS-Blast against the National Center for Biotechnology Information conserved domain database (CDD). Error bars represent standard deviation

The 28 cocci-shaped *Sporosarcina* strains sequenced in the study and the previously sequenced *S. ureae* str. DSM 2281, revealed some general genomic characteristics of the cocci-shaped *Sporosarcina*. The average cocci-shaped *Sporosarcina* genome is 3.33 Mb in size with a GC content range of 41.7–44.0%, encoding for an average 3222 CDS, 54 tRNAs and six ribosomal loci (Table 1), in comparison to the closely related bacillus-shaped *S. newyorkensis* that has a predicted slightly larger average genome of 3.61 Mb encoding 3673 CDS or over 450 more genes. To establish a general functional role of the 3222 CDS present in the average genome, the clusters of orthologous groups of proteins (COGs) for each of the genomes were determined. On average, 89.2% CDS (2874 out of 3222) could be assigned to a COG category for the 29 cocci-shaped *Sporosarcina* genomes (Fig. 3). Excluding categories R (General function prediction only) and S (Function unknown) the top three COG categories were E (Amino acid transport and metabolism), K (Transcription), and P (Inorganic ion transport and metabolism), while the lowest three with at least two genes were D (Cell cycle control, cell division, chromosome partitioning), V (Defense mechanisms), and U (Intracellular trafficking, secretion, and vesicular transport).

Presence of mobile genetic elements was variable between the genomes depending on the type of element. Only strains P35 and P37 were found to contain a plasmid. Whereas, the cocci-shaped *Sporosarcina* genomes ranged from 2 to 46 insertion sequence (IS) elements, with an average of 15.6 per genome. The amount of IS elements present in a genome were widely variable among the different strains, S204 had 46 IS elements, while the closely related type strain DSM 2281 only had 2 IS elements. Moreover, the genomes of the closely related strains, P33, P35 and P37, had 29, 16 and 37 IS elements, respectively. Although only having draft genomes might be having a slight effect on the analysis, those with complete genomes still ranged from 12 to 46 IS elements. Additionally, the cocci-shaped *Sporosarcina* genomes contain a range of 1 to 6 prophages with an average of 2.3 per genome, however the vast majority were incomplete prophages. The cocci-shaped *Sporosarcina* genomes demonstrate a significant amount of synteny, although seven strains (P10, P12, P19, DSM 2281, S204, and P18a) contain a single ~ 791 kb inversion located between 1,498,920 and 2,290,700 (S204 genome coordinates). The inversion appears to be due to the result of mobile genetic elements, as it contains an ISSpglI element (*Sporosarcina globispora*) on both ends (Additional file 2: Figure S2).

### Analysis of methylome

A subset of six cocci-shaped *Sporosarcina* strains that were sequenced exclusively with PacBio sequencing technology allowed for methylome analysis. This analysis revealed that at the epigenetic level there are significant differences among these six genomes, as only one shared DNA methylation motif (TTCGGA), between P33 and P37, was identified between the genomes under the growth conditions used for the study. Interestingly, S204 is the only strain to contain a Dam methylation motif, which also happened to be the only methylated motif throughout the genome. Strains P17a, P32a, and P33 each contain multiple methylated motifs including both m6A and m4C methylation, while P37 lacks any apparent cytosine methylation. Additionally, P8 has no currently identified adenine or cytosine methylation motifs, but did have several base modifications of unknown types (Table 2; Additional file 3: Figure S3). The different phylogenetic analyses used throughout the study places these six strains in five different clades, suggesting some genomic diversity among these strains, which is further supported by high level of variation at the epigenetic level. However, even those that belong in the same clade (P33 and P37) and contain the only shared methylation motif, variation is still seen in number of motifs: P37 had three motifs methylated, while P33 had nine.

**Table 2:** Methylation profiles of six strains of cocci-shaped *Sporosarcina*

Recognition Sequence	Type/ subtype	Unique	% Detected	Coverage	Potential Methylases	Methylation Type
*Sporosarcina* sp. P17a						
BCGCCGANRD	II	yes	50.6	90.6		
CCGYAG	II	yes	100	90.5	SurP17aORF5150P	6 mA
CGCCGTTNNNB	II	yes	21.4	89.2		
CGCCGVNY	II	yes	59.3	93.2		
CGGCGNYD	II	yes	42.5	91.5		
CGSCGNBV	II	yes	18.3	84.9		
GCGGTAVYR	II	yes	21	92.2		
TGAAATT	II	yes	99.9	82.5	SurP17aORF5155P	6 mA
*Sporosarcina* sp. P32a						
CCAG	II	no	30.9	74.5		
CAAYNNNNNGTAA	I gamma	yes	100	80.6	M.SurP32aI	6 mA
ACRGAG	II G,S,gamma	yes	100	82.4	SurP32aII	6 mA
*Sporosarcina* sp. S204						
GATC	II	no	29.2	84.2		6 mA
*Sporosarcina* sp. P8						
CGTCGANA	II	yes	73.9	351.8		
CGTCGTNGD	II	yes	21.2	363.7		
CGTCGTNYR	II	yes	76.8	350.6		
CGTCGTTNY	II	yes	44.8	356.2		
CGWCGVNB	II	yes	69.2	355.3		
DNGCCACNCA	II	yes	23.5	365.9		
GGGGCATNNNNNNNH	II	yes	16.9	341.2		
*Sporosarcina* sp. P37						
GACGAG	II	no	99.6	72.8	SspP37ORF15190P	6 mA
GCCATC	II	no	100	73.2	M.SspP37ORF13670P	6 mA
TTCGAA	II	no	100	71.7	M.SspP37I	6 mA
*Sporosarcina* sp. P33						
ACGNNNNNNTAYNG	I	yes	100	84.6		
ANCDGGGAC	II	yes	28.4	82.7		
DNCGCGGTANY	II	yes	26.5	86.3		
GGHANNNNNNTTTA	I	yes	99.8	84.7		
GTCCCBVNY	II	yes	52.2	87.6		
GTCCCGBANNNNNNH	II	yes	29.4	85.9		
SGTCCCNY	II	yes	23.2	85.6		
TTCGAA	II	no	100	81.7	M.SspP33I	6 mA
GGGAC	II	no	100	84.8	SspP33II	6 mA

### Comparative genomic analysis of cocci-shaped *Sporosarcina* strains

**Fig. 4:**
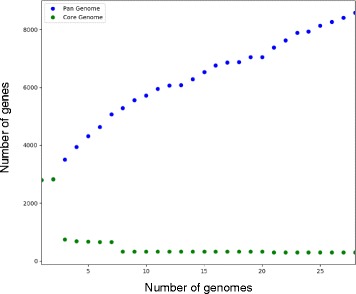
The core and pan-genomes of cocci-shaped strains of *Sporosarcina*. Using BLASTp, and cutoff values of 90% amino acid identity across 90% of the gene, 221 conserved core genes were identified among the strains. The pan genome has 8610 genes using 30% identity across 70% of the gene as cutoff parameters

**Fig. 5:**
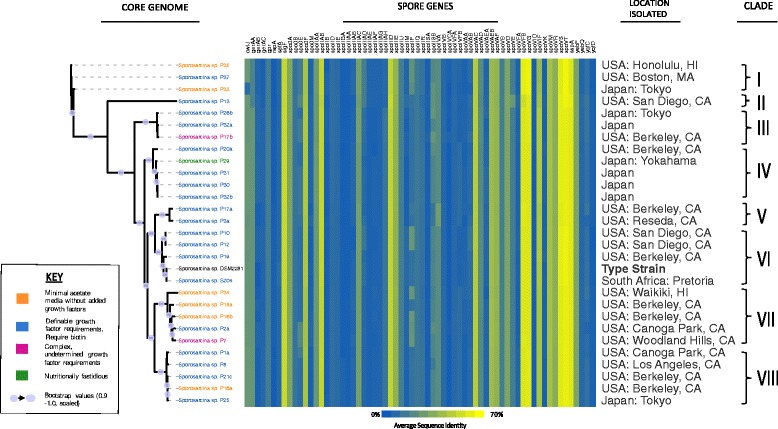
Phylogenetic tree of the cocci-shaped *Sporosarcina* strains based on the core genome, and rooted based on the 16S rRNA gene tree. Tree was built using core genes shared at 90% amino acid sequence identity and 90% sequence coverage by all strains. Those sequences were concatenated in the same order for each genome, aligned using MAFFT, and the tree was built using FastTree. The genome names are colored to reflect the phenotypic class they were assigned during their initial isolation by Pregerson (1973). Spore genes found in *B. subtilis* and other spore-forming bacteria were protein-blasted to determine whether similar sequences exist in cocci-shaped strains of *Sporosarcina*

Based on the initial diversity observed between the cocci-shaped *Sporosarcina* strains at the 16S rRNA gene level, we investigated how that diversity held up at the genomic level by utilizing whole genome sequence (WGS) analysis to reveal higher levels of resolution between stains. The pan-genome analysis of the 28 sequenced cocci-shaped *Sporosarcina* strains plus the previously sequenced *S. ureae* str. DSM 2281 using 30% identity cutoff contains a total of 8610 genes. Interestingly, core genome analysis of all 29 cocci-shaped *Sporosarcina* strains at a 90% identity cutoff established only 221 core genes or only about 7% of the total genome (Fig. 4). Overall, the presumably more reliable and higher resolution of a phylogenetic analysis based on the identified core genome placed the 29 cocci-shaped *Sporosarcina* strains into eight clades, which was down from the 11 clades from the 16S rRNA gene level analysis (Fig. 5). The cocci-shaped *Sporosarcina* genomes averaged 57 strain-specific genes, but the amount varied widely. Strains that lacked a very close phylogenetic neighbor tended to have a larger pool of strain-specific genes, for example strain P13, which forms its own clade, has 213 strain-specific genes or 6.6% of the genome. Phylogenetic analysis based on the accessory genes generated a phylogenetic relationship that was nearly identical to the core genome as it also separated the strains into eight different clades. All strains were also placed in the exact eight clades, but there were minor modifications as to the relationship within the clade (Additional file 4: Figure S4). Furthermore, the core and accessory gene phylogenetic relationships failed to cluster strains based on isolation location or Pregerson’s original nutritional requirement grouping phenotypes.

Sporulation is a key characteristic of the genus *Sporosarcina*, therefore, to further understand the diversity between the cocci-shaped *Sporosarcina* strains we examined spore-related genes. Examining cocci-shaped *Sporosarcina* genomes for 66 spore-related genes present in *Bacillus subtilis*, revealed that many of these genes are actually missing. Nevertheless, the overall presence or absence of these sporulation genes in cocci-shaped *Sporosarcina* strains was fairly well conserved across all the strains (Fig. 5). However, the amino acid identity between the strains did have some variation, as phylogenetic analysis of the 29 cocci-shaped *Sporosarcina* strains based on the spore-related genes generated a tree nearly identical to the core genome tree. In fact, it was identical to the phylogenetic tree generated by the accessory genes, and placed the strains into the exact same eight clades as both the core genome and accessory. The sporulation gene tree also generated the same shifts in the relationships between strains within the same clades as the accessory gene tree (Data not shown).

### Potential novel cocci-shaped *Sporosarcina* species

**Fig. 6:**
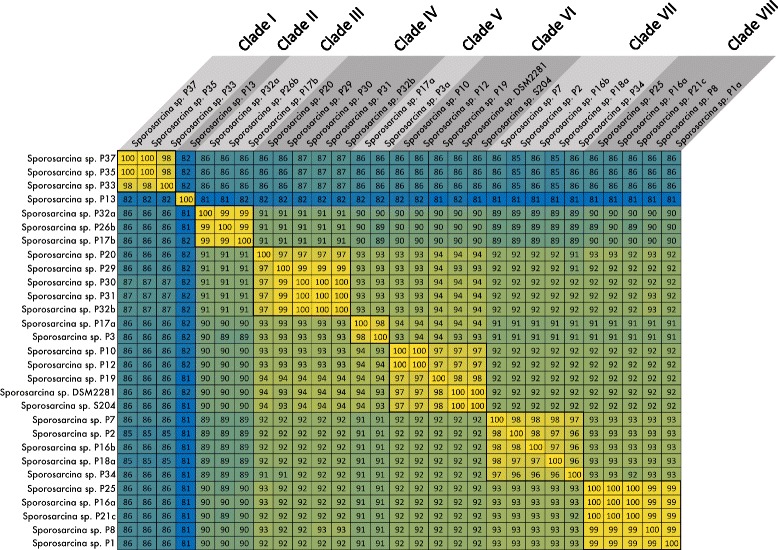
Average amino acid identity matrix between the 29 cocci-shaped *Sporosarcina* strains sequenced during this study. Thick black boxes indicate species 95-96% cutoffs as proposed by Konstantinidis and Tiedje (2005), and were generated with the Genome-based distance matrix calculator website

Since the phylogenetic relationship based on core genes, accessory genes and spore-related genes all indicate that these 29 cocci-shaped *Sporosarcina* strains should be separated into eight different clades, we examined if these were potentially novel species of cocci-shaped *Sporosarcina* based on the average amino acid identity (AAI) between strains (Fig. 6). Strains within clade 1 (P35, P33 and P37) share 99.3% AAI, but only 82% AAI with the clade 2 (P13) and 86% AAI with clade 8 (P1a, P8, P21c, P16a, and P25). Whereas, strains within clade 7 (P7, P2, P16b, P18a, and P34) share 97.1% AAI, but only 89% AAI with clade 3 (P26b, P32a, and P17b). Furthermore, clade 6 (P10, P12, P19, S204 and DSM 2281) that includes the *S. ureae* type strain (DSM 2281) share 97.8% AAI between the strains, but just 93.7% AAI with the nearest neighbor clade 5 (P17a and P3a). Overall, all the strains within a clade share the 95% AAI minimum for identical species, but none of the clades share the 95% minimum between them (Fig. 6).

**Fig. 7:**
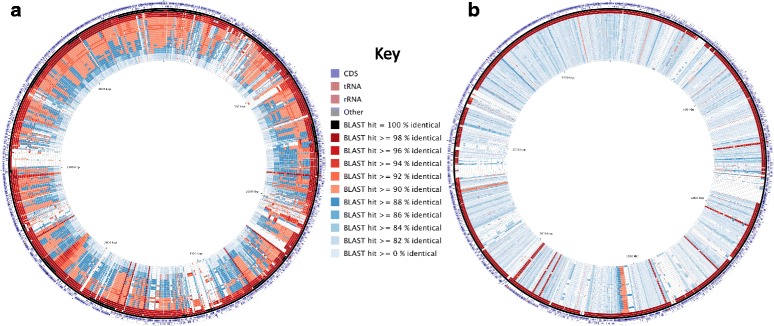
BLAST Atlases comparing the 29 cocci-shaped *Sporosarcina* genomes against one of two complete reference genomes (S204 and P33), circular plots were generated with CGView Comparison Tool using BLASTn. Genomes are arranged with the genetically closest to reference genome on the outer ring, and most distantly related on the inner most ring. **a** S204 reference genome; ordered from outer ring to inner ring: 1) Forward CDS, tRNA and rRNA; 2) Reverse CDS, tRNA and rRNA; 3) S204; 4) DSM 2281; 5) P19; 6) P12; 7) P10; 8) P29; 9) P31; 10) P30; 11) P32b; 12) P17a; 13) P20a; 14) P3; 15) P1; 16) P21c; 17) P16a; 18) P25; 19) P18a; 20) P2; 21) P8; 22) P16b; 23) P7; 24) P34; 25) P26b; 26) P32a; 27) P17b; 28) P37; 29) P35; 30) P33; 31) P13. **b** P33 reference genome; ordered from outer ring to inner ring: 1) Forward CDS, tRNA and rRNA; 2) Reverse CDS, tRNA and rRNA; 3) P33; 4) P37; 5) P35; 6) P12; 7) P10; 8) P17a; 9) P31; 10) P30; 11) P29; 12) P32b; 13) P19; 14) S204; 15) P3; 16) P17b; 17) P26b; 18) P20a; 19) P32a; 20) DSM 2281; 21) P34; 22) P21c; 23) P8; 24) P16a; 25) P25; 26) P1; 27) P16b; 28) P18a; 29) P7; 30) P2; 31) P13

Furthermore, to investigate the average nucleotide identity (ANI) variation between the different clades, and also further the analysis at the DNA level, BLAST atlases using each strain as a reference were produced using the BLASTn command to compare each of the genomes against the reference genome (Fig. 7). For visualization only those strains with a complete genome were utilized as a reference genome, therefore setting S204 as the reference demonstrates that only the strains present in clade 6 (P10, P12, P19, S204 and DSM 2281) share ≥94% ANI across the vast majority of the genome. Whereas, all the remaining 24 strains share ≤92% ANI with S204 and the other strains present in clade 6. On the other hand, applying P33, a member of clade 1 (P33, P35, and P37), as the reference genome found it shared ≥96% ANI with the other strains in the clade. However, all 26 other cocci-shaped *Sporosarcina* strains share ≤86% ANI with P33, which also demonstrates at the DNA level the diversity between the eight different clades. Overall, the AAI and ANI variability between the strains present in the eight different clades suggests these clades may represent novel species of cocci-shaped *Sporosarcina* strains.

## Discussion

Members from the genus *Sporosarcina* have been isolated from very diverse environments such as soil [39], food production facility [40], or clinical samples [41] just to name a few. In the 1970s, Pregerson isolated over 50 cocci-shaped *Sporosarcina* strains from three different continents, and found they were most commonly isolated from soils exposed to human or animal urine [12]. However, no study has collective examined the general global distribution of the genus *Sporosarcina*, or the most common environment associated with members. Investigating the geographic distribution of the genus *Sporosarcina* showed, similar to the cocci-shaped *Sporosarcina* strains, the other species have a global dissemination. Furthermore, the genus could be found in terrestrial, human, animal, and plant environments, but was most commonly associated with terrestrial colonization, again similar to the soil associated cocci-shaped *Sporosarcina* strains. It may be that other environments such as plants or animals get colonized through soil contamination, but additional surveillance studies are needed to determine for sure. Ultimately, the global distribution of cocci-shaped *Sporosarcina* strains appears to be similar to the genus *Sporosarcina* as a whole.

Phylogenetic relatedness based on the 16S rRNA gene indicates that these cocci-shaped *Sporosarcina* strains including *S. ureae* belong in the genus with the other bacilli-shaped *Sporosarcina* species. The 16S rRNA gene analysis predicts the closest neighbor is the bacilli-shaped *S. newyorkensis*, but it also confirmed the diversity of the cocci-shaped *Sporosarcina* strains predicted from previous studies, as it divided the 28 sequenced strains, DSM 2281, and four additional *S. ureae* 16S rRNA sequences into 11 clades. However, analysis failed to find a direct correlation between distance isolated and 16S rRNA gene similarity. Additionally, using the single 16S rRNA gene did not provide the resolution needed to decipher the phylogenetic relationships of the cocci-shaped *Sporosarcina* strains. For example, organisms that share at least 97% pairwise identity at the 16S rRNA gene level, along with other phenotypic markers, are considered the same species [42]. Nonetheless, *S. newyorkensis* shares 98.1% pairwise identity with P33 and P37, and 97.2% pairwise identity with P13, although they are lacking the phenotypic markers, at the 16S rRNA gene level it suggests they are the same species. In fact, it is not until the distant clades including *S. ureae* DSM 2281 16S rRNA gene identity drops below the 97% level (96.9% pairwise identity) with *S. newyorkensis*, but current research has suggested moving the cutoff to 98.65% particularly when combined with other genomic metrics such as ANI, might clarify the process of distinguishing novel species [43]. In fact, using 98.65% would resolve the issue between the cocci-shaped *Sporosarcina* strains and *S. newyorkensis*. Moreover, many of the cocci-shaped *Sporosarcina* strains would also not be considered the same species as *S. ureae*, which supports the comparative genomic analysis data too. Notwithstanding, the analysis does suggest that the cocci-shaped *Sporosarcina* strains P33, P37, and P35 are closely related to the bacilli-shaped *S. newyorkensis*, thus future comparative genomic studies could help resolve how the cocci-shaped strains fit in the genus, as well as a genetic resource for investigating cell morphology and sporulation in cocci-shaped bacteria.

The diversity predicted by the 16S rRNA gene analysis, nutritional growth requirement analysis, and enzymological analysis were also supported by methylome analysis under the growth conditions utilized in this study. In fact, no motif was common to all six strains and only one motif (TTCGAA) of 31 determined by PacBio sequencing, is shared between P33 and P37. Interestingly, both of these strains, which share 99% AAI across the entire genome still contain substantial variation in their methylomes. Moreover, P33 and P37 have different phenotypes as they were grouped into different nutritional groups by Pregerson, which suggests that methylome difference could result in gene expression differences in the strains [44]. We hypothesize that these variations in the methylome allow the closely related cocci-shaped *Sporosarcina* strains to adapt to different environments or slightly different ecological niches, as these two strains were isolated on opposite sides of the world. Future work, such as epigenomic and transcriptomic studies of the various cocci-shaped strains, is needed to completely address the role DNA methylation has in variation of phenotype.

The study found that on average a cocci-shaped *Sporosarcina* strain contains a 3.3 Mb genome that encodes for 3222 CDS, approximately 11.1% of those CDS have only a general function predicted, 8.9% CDS an unknown function, and 8.7% CDS for amino acid transport and metabolism. In the soil, nitrogen is very limited for plants, bacteria and other microbes, therefore there is a high level of competition for available nitrogen. Ammonium is a preferred form of nitrogen for many soil bacteria and fungi [45], however amino acids, such as glutamine and glutamate, are another critical source of nitrogen for bacteria [46]. Cocci-shaped *Sporosarcina* strains contain urease that can break urea down into ammonia when it is available, and probably represents a major method of nitrogen acquisition for these strains based on Pregerson isolating from soils with frequent urine exposure. Yet it is possible that the high level of amino acid transport and metabolism genes are present as a backup system to acquire critical nitrogen from the soil environment in the absence of urea. Again, future work examining the role urease and amino acid acquisition has among the cocci-shaped *Sporosarcina* strains survival in different types of soils is needed to directly answer these questions.

The exact role mobile genetic elements have in the diversity of cocci-shaped *Sporosarcina* strains is unclear, particularly since the presence or absence of certain types of mobile genetic elements were quite variable. For example, out of the 29 genomes analyzed during this study only the closely related strains P37 and P35 contained a plasmid. Additionally, on average there were 2.3 prophages per genome, but those were almost always incomplete or prophage scars, as only 27.6% (8 out of 29) of the genomes contained an intact prophage. There was definitely no evidence of large fluctuations in the genome size due to the presence or absence of prophage sequences, particularly like *Escherichia coli* where the prophages are a critical evolutionary driver and cause massive changes to the genome size [47]. However, there was a lot of variability with the amount of IS elements present in the various genomes of cocci-shaped *Sporosarcina* strains, and IS elements are also a known driver of *E. coli* evolution particularly O157:H7 [48]. In fact, the study found a role in the cocci-shaped *Sporosarcina* evolution, as there is fairly strong synteny among most of the strains, except for seven strains that contain an approximately 791 kb (~ 24% of the genome) inversion that is due to the presence of ISSpglI elements (*Sporosarcina globispora*) on each end of the inversion. Yet, the exact role mobile genetic elements particularly IS elements play in the evolution and diversity of cocci-shaped *Sporosarcina* strains will need particular in-depth analysis beyond the scope of this current study.

Sporulation is a key characteristic of the genus *Sporosarcina* including the cocci-shaped strains, but analysis with 66 spore-related genes from *Bacillus subtilis* found that at ≥20% AAI cocci-shaped *Sporosarcina* strains only contained 38% (25 out of 66) of those genes. Yet, those 25 spore-related genes are well conserved among all 29 strains of cocci-shaped *Sporosarcina*, nonetheless, the other 41 spore-related genes have AAI variation among the cocci-shaped *Sporosarcina*. These 41 spore-related genes may be critical drivers of the diversity of these strains, as phylogenetic relatedness analysis based on all 66 of the spore-related genes generated a phylogenetic tree nearly identical to that of the core genome. It is possible that there are novel spore-related genes not identified in this study, but it does provide a framework for future work investigating sporulation in cocci-shaped bacteria.

Inferring bacterial relationships based on whole genome DNA sequences is a difficult endeavor due to the vast amount of sequence shared during horizontal gene transfer (HGT). To counter this, studies indicate that using a smaller subset of “core” genes would minimize the effect of HGT skewing phylogenetic analysis [49]. While there are multiple methodologies to generate a core genome, a consensus as to what defines a core genome does not exist. Furthermore, using WGS data to define novel species also still under debate, but Konstantinidis and Tiedje determined that the 70% DDH species cutoff corresponds with an average amino acid identity of 95-96% [38, 50]. Thus, using a series of highly conserved genes, such as those found in a core genome, we can resolve the phylogenetic relationships of very closely related strains and identify novel species among those strains. Utilizing a 90% AAI cut-off generated a core genome of 221 genes or 7% of the genome among the cocci-shaped *Sporosarcina* strains. Using a lower 75% AAI cut-off expands the core genome to 881 genes or 27.3% of the genome. However, these are both lower than other described core genomes, for example Rasko et al. used an 80% AAI cut-off and found *E. coli* had a core genome of 2344/5020 or 46.7% of the genome [51]. While, using a 90% cut-off, Leekitcharoenphon et al. found *Salmonella enterica* had a core genome of 2882 genes or approximately 64% of the genome [52]. Again, this low level of core genes further supports the large amount of genomic diversity among these cocci-shaped *Sporosarcina* strains. Ultimately, the higher resolution provided by WGS and comparative genomics refined the 29 cocci-shaped *Sporosarcina* strains done from the 11 clades predicted by 16S rRNA gene analysis to just eight clades. In fact, phylogenetic relatedness predicted by core gene, accessory gene or spore-related gene analysis all place the strains into the exact same eight clades. In fact, using the Konstantinidis and Tiedje suggested 95% AAI cut-off for species, only those strains clustered within a common clade would be the same species. Additionally, clade 1 (P33, P35, and P37) only has 85.9% AAI and clade 2 (P13) only 81.5% AAI to the other cocci-shaped *Sporosarcina* strains, supporting that both clades comprise novel cocci-shaped *Sporosarcina* species. Again, all these results support Pregerson’s result from the original 1973 phenotype study of these strains, as she predicted that the cocci-shaped *Sporosarcina* strains isolated from around the world were quite diverse.

As more genomes are becoming sequenced, the definition of what constitutes a prokaryotic species is being challenged. Until recently, a prokaryotic species was defined as a strain (including the type strain) characterized by certain phenotypic consistency, 70% DNA-DNA hybridization (DDH) and over 97% identity of the 16S rRNA gene [38]. With the advent of affordable whole genome DNA sequencing (WGS), the ability to study organisms at the individual nucleotide level allows for refining phylogenetic relationships that were originally based on the classic polyphasic approach. There currently exists a push to include parameters derived from whole genome sequencing, such as average nucleotide identity or average amino acid identity to delineate species [53–55]. One such study used ANI and alignment fraction to calculate the probability that two genomes belong to the same species, showing these metrics are often far more accurate than DDH and 16S rRNA identity. Moreover the same study shows these metrics will help reclassify organisms that currently have the same taxonomic classification, but cluster separately based on genomic metrics [56]. In this study, we show that 29 strains of cocci-shaped *Sporosarcina* are much less related to each other than the polyphasic metrics would suggest. Although it has been suggested for a long time, Hug et al. demonstrated that using more genes resolved phylogenetic relationships particularly those that were more ambiguous when using just one gene [57].

## Conclusions

In conclusion, this is the first study to investigate the genomics of not just cocci-shaped *Sporosarcina*, but any species of the genus *Sporosarcina*, in fact, the study more than tripled the amount of WGS sequence data available for the genus *Sporosarcina*. During this study, genomes of 28 cocci-shaped strains of the genus *Sporosarcina* were sequenced and characterized, and comparative genomics of these cocci-shaped strains isolated from around the world revealed a high level of diversity. In fact, we have shown that, although they share morphological, biochemical, and 16S rDNA similarity, they are remarkably variable in their gene content, genome sequence identity, and methylomes. Based on the phylogenetic relationship generated from either core genes, accessory genes or spore-related genes these cocci-shaped *Sporosarcina* strains are always divided into eight different clades, thus suggesting there may be up to seven novel cocci-shaped *Sporosarcina* species in addition to *S. ureae*. Although it requires additional phenotypic analysis to confirm these different clades, based on the strong AAI and ANI variation among the strains, we conclude that clade 1 (P33, P35, and P37) and clade 2 (P13) represent new species.

## Additional files

Additional file 1: **Figure S1.** Correlation of the pairwise distance isolated compared to 16S rRNA gene similarity. All sequence information was retrieved from the Earth Microbiome Project or Genbank. Red line indicates an R value of 0.0147. (PPTX 178 kb)

Additional file 2: **Figure S2.** ACT (Artemis Comparison Tool) alignment plot of strains of cocci-shaped *Sporosarcina.* Bands indicated shared genes. Red bands are genes shared in the same direction and blue bands are genes share in reverse directions (sequence inversions). (PPTX 6951 kb)

Additional file 3: **Figure S3.** Circos plot showing type and location of DNA methylation modifications of six strains of *Sporosarcina* that were sequenced with Pacific Biosciences technology*.* Color of lines indicate type of modification: adenine (blue), cytosine (red), and unknown (yellow). The lower table is a key for each ring present on the circos plot. (PPTX 23969 kb)

Additional file 4: **Figure S4.** The phylogenetic tree is based on the core genome, and the matrix displays the presence or absence of genes in blue and white respectively, for each strain, in the pan-genome. At 30% amino acid sequence idenity, across 70% of the gene, there are 8610 unique gene clusters that make up the pan-genome of the 29 *Sporosarcina* strains. (PPTX 15892 kb)

## Abbreviations

AAI: Average amino acid identity
ACT: Artemis Comparison Tool
ANI: Average nucleotide identity
BLAST: Basic Local Alignment Search Tool
CDD: Conserved Domain Database
CDS: Coding DNA sequence
COG: Cluster of Orthologous Groups
DDH: DNA-DNA hybridization
EMP: Earth Microbiome Project
GEOS: Geometry Engine – Open Source
HGT: Horizontal gene transfer
IS: Insertion sequence
ISSpglI: Insertion sequence of *Sporosarcina globispora*
iTOL: Interactive Tree of Life
NCBI: National Center for Biotechnology Information
PC: Percent query coverage
PGAAP: Prokaryote Genome Automatic Annotation Pipeline
PI: Percent amino acid sequence identity
QV: Quality Value
RDP: Ribosomal Database Project
RPS-BLAST: Reverse Position Specific Basic Local Alignment Search Tool
SMRT: Single Molecule Real Time Sequencing
WGS: Whole genome sequencing
