# Supplementary figures and images for: Comparative genomics of cocci-shaped Sporosarcina strains with diverse spatial isolation
Source: BMC Genomics. 2018 May 2;19:310. doi: 10.1186/s12864-018-4635-8 (PMC5930826; doi:10.1186/s12864-018-4635-8)

## Slide 1
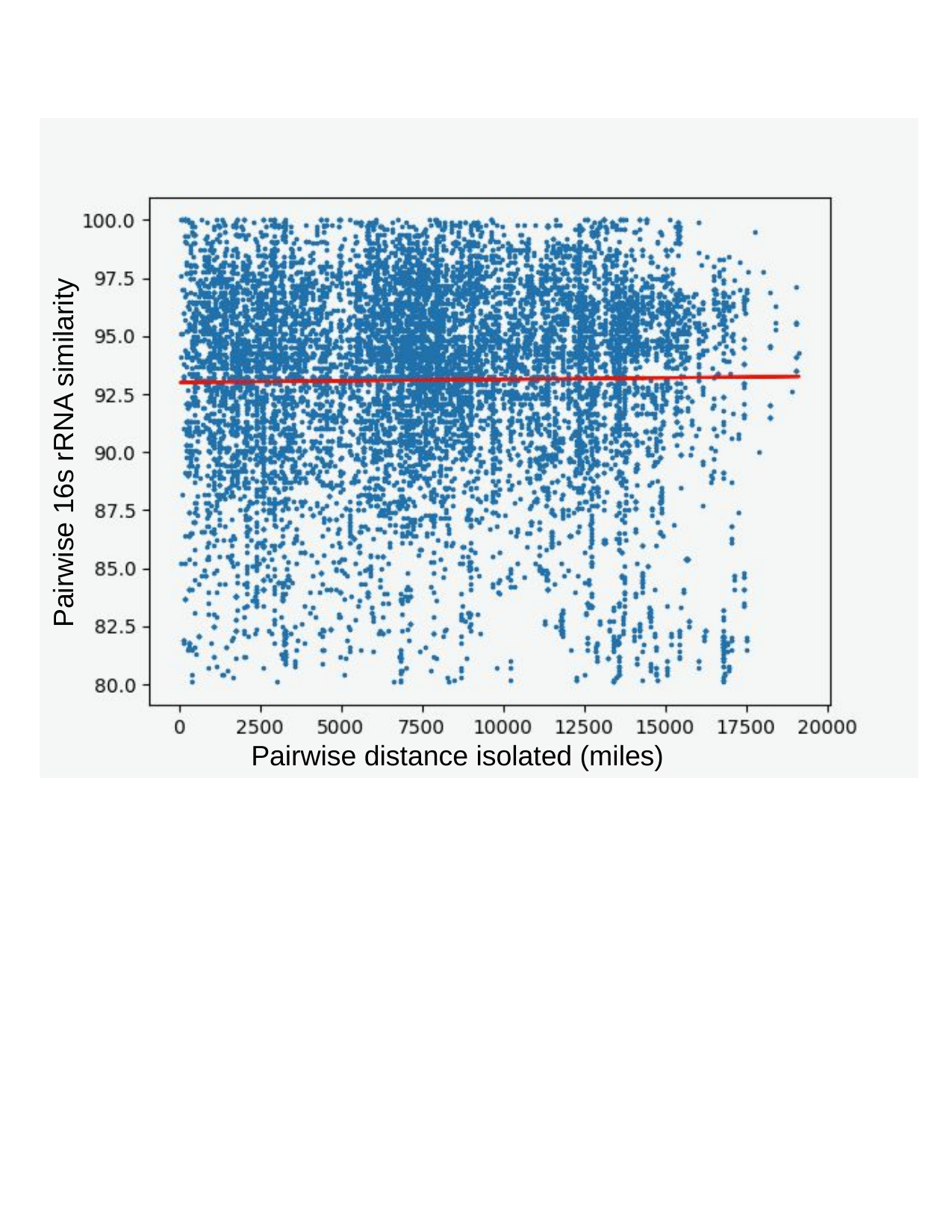

Pairwise 16s rRNA similarity
Pairwise distance isolated (miles)

Supplement: Supplementary file 1 — Figure S1. Correlation of the pairwise distance isolated compared to 16S rRNA gene similarity. All sequence information was retrieved from the Earth Microbiome Project or Genbank. Red line indicates an R value of 0.0147. (PPTX 178 kb) [file 12864_2018_4635_MOESM1_ESM.pptx]

## Slide 1
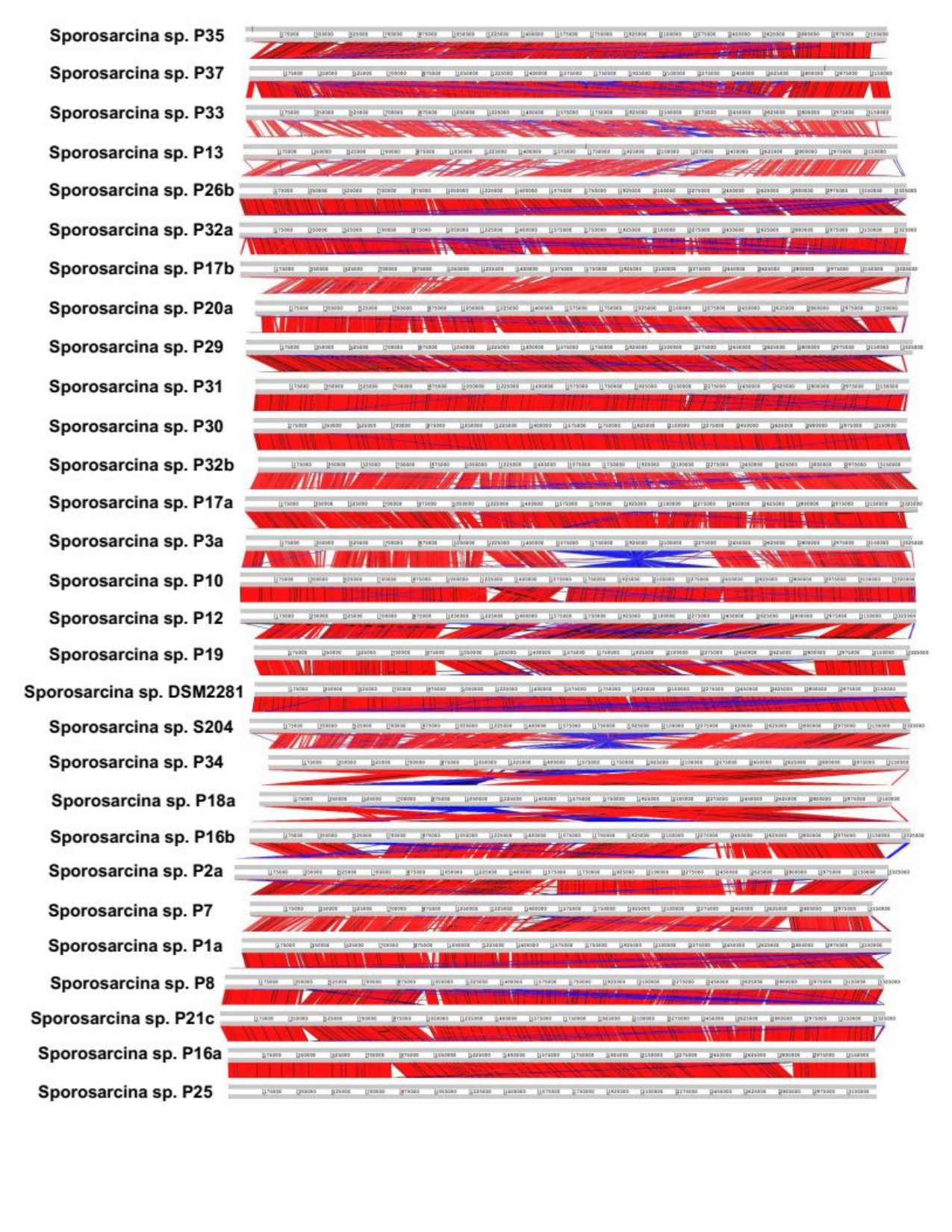

Supplement: Supplementary file 2 — Figure S2. ACT (Artemis Comparison Tool) alignment plot of strains of cocci-shaped Sporosarcina. Bands indicated shared genes. Red bands are genes shared in the same direction and blue bands are genes share in reverse directions (sequence inversions). (PPTX 6951 kb) [file 12864_2018_4635_MOESM2_ESM.pptx]

## Slide 1
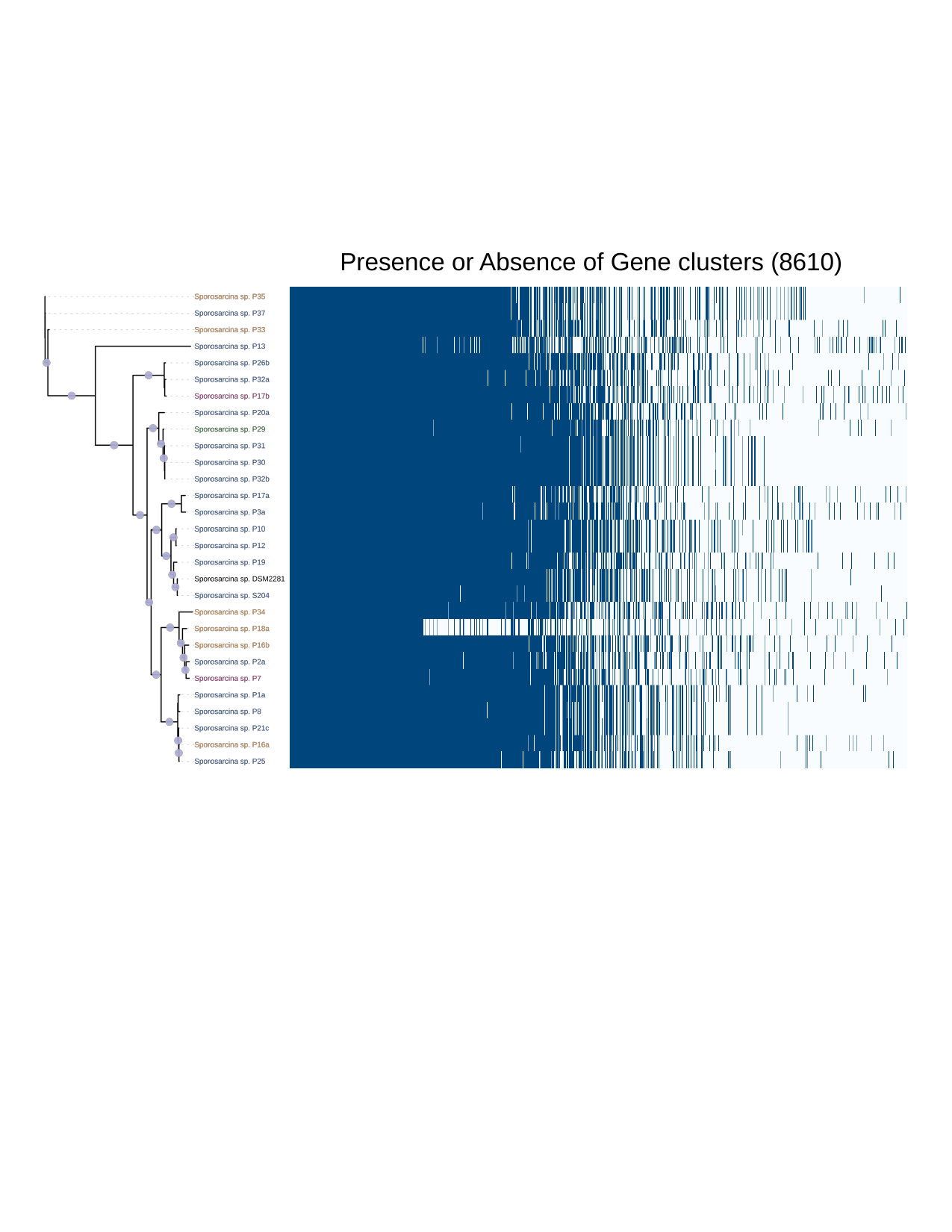

Presence or Absence of Gene clusters (8610)

Supplement: Supplementary file 4 — Figure S4. The phylogenetic tree is based on the core genome, and the matrix displays the presence or absence of genes in blue and white respectively, for each strain, in the pan-genome. At 30% amino acid sequence idenity, across 70% of the gene, there are 8610 unique gene clusters that make up the pan-genome of the 29 Sporosarcina strains. (PPTX 15892 kb) [file 12864_2018_4635_MOESM4_ESM.pptx]
