# Supplementary material for: Comparative genomics of cocci-shaped Sporosarcina strains with diverse spatial isolation
Source: BMC Genomics. 2018 May 2;19:310. doi: 10.1186/s12864-018-4635-8 (PMC5930826; doi:10.1186/s12864-018-4635-8)
Supplement: Supplementary file 3 — Figure S3. Circos plot showing type and location of DNA methylation modifications of six strains of Sporosarcina that were sequenced with Pacific Biosciences technology. Color of lines indicate type of modification: adenine (blue), cytosine (red), and unknown (yellow). The lower table is a key for each ring present on the circos plot. (PPTX 23969 kb) [file 12864_2018_4635_MOESM3_ESM.pptx]

## Slide 1
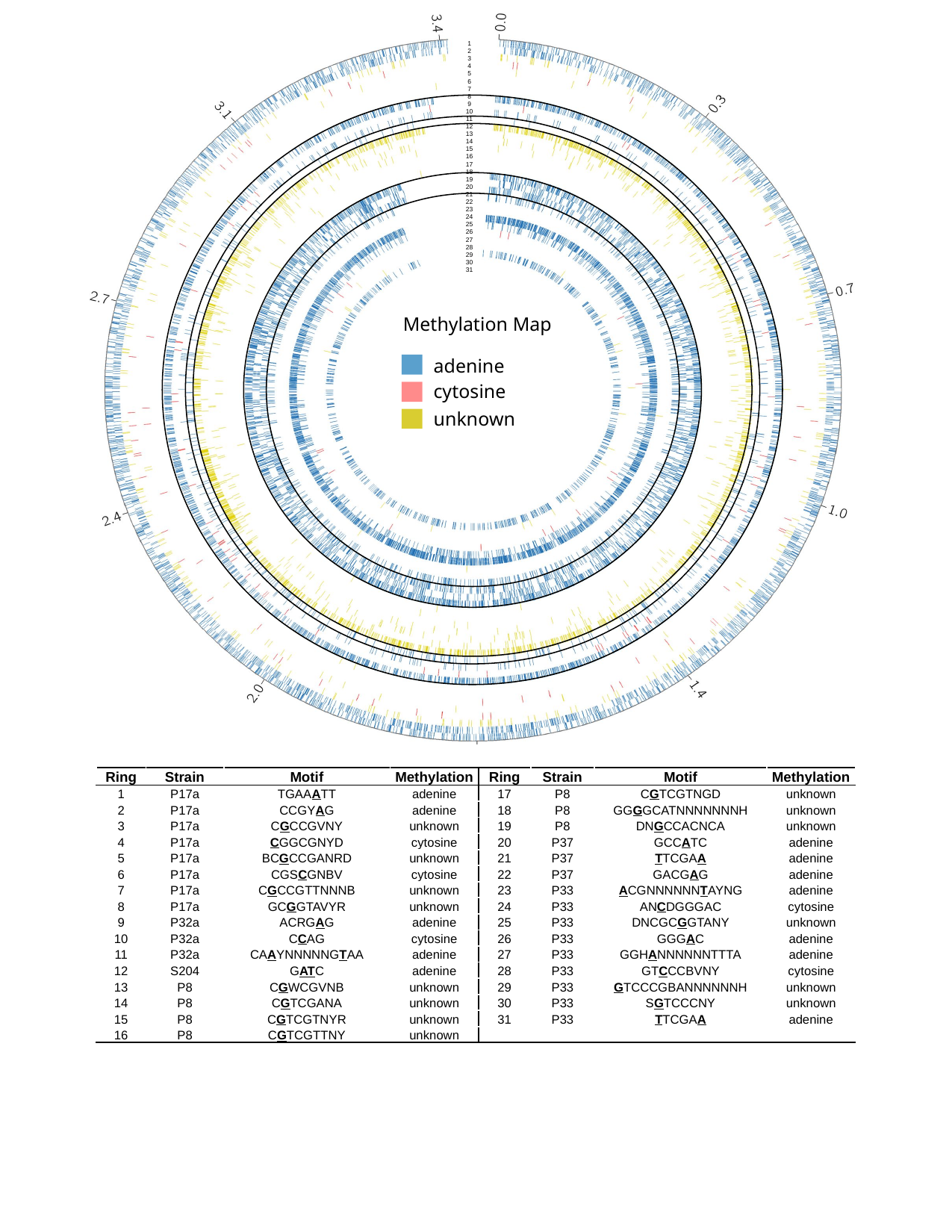

1
2
3
4
5
6
7
8
9
10
11
12
13
14
15
16
17
18
19
20
21
22
23
24
25
26
27
28
29
30
31
Methylation Map
adenine
cytosine
unknown
| Ring | Strain | Motif | Methylation | Ring | Strain | Motif | Methylation |
| --- | --- | --- | --- | --- | --- | --- | --- |
| 1 | P17a | TGAAATT | adenine | 17 | P8 | CGTCGTNGD | unknown |
| 2 | P17a | CCGYAG | adenine | 18 | P8 | GGGGCATNNNNNNNH | unknown |
| 3 | P17a | CGCCGVNY | unknown | 19 | P8 | DNGCCACNCA | unknown |
| 4 | P17a | CGGCGNYD | cytosine | 20 | P37 | GCCATC | adenine |
| 5 | P17a | BCGCCGANRD | unknown | 21 | P37 | TTCGAA | adenine |
| 6 | P17a | CGSCGNBV | cytosine | 22 | P37 | GACGAG | adenine |
| 7 | P17a | CGCCGTTNNNB | unknown | 23 | P33 | ACGNNNNNNTAYNG | adenine |
| 8 | P17a | GCGGTAVYR | unknown | 24 | P33 | ANCDGGGAC | cytosine |
| 9 | P32a | ACRGAG | adenine | 25 | P33 | DNCGCGGTANY | unknown |
| 10 | P32a | CCAG | cytosine | 26 | P33 | GGGAC | adenine |
| 11 | P32a | CAAYNNNNNGTAA | adenine | 27 | P33 | GGHANNNNNNTTTA | adenine |
| 12 | S204 | GATC | adenine | 28 | P33 | GTCCCBVNY | cytosine |
| 13 | P8 | CGWCGVNB | unknown | 29 | P33 | GTCCCGBANNNNNNH | unknown |
| 14 | P8 | CGTCGANA | unknown | 30 | P33 | SGTCCCNY | unknown |
| 15 | P8 | CGTCGTNYR | unknown | 31 | P33 | TTCGAA | adenine |
| 16 | P8 | CGTCGTTNY | unknown | | | | |
